# Control of *MSSA and MRSA* in the United States: protocols, policies, risk adjustment and excuses

**DOI:** 10.1186/s13756-019-0550-2

**Published:** 2019-06-19

**Authors:** Kevin T. Kavanagh

**Affiliations:** Health Watch USA, Lexington, KY USA

**Keywords:** MRSA, MSSA, CDC, Surveillance, Isolation, Veterans health administration, VA, Standards, SIR, Risk adjustment

## Abstract

Data released by the U.S. Centers for Disease Control and Prevention (CDC) on March 5, 2019 showed that *Staph aureus* infections are a major problem in the United States, with 119,000 infections and almost 20,000 deaths in 2017. Rates of decline for hospital-onset MRSA have slowed since 2012 and the United States is not on track for meeting the 2015 U.S. Dept. of Health and Human Services’ goal of a 50% reduction by 2020. There is a need for improved standards for control of dangerous pathogens. Currently, the World Health Organization’s recommendation of preoperatively screening patients for *Staph aureus* has not become a standard of care in the United States.

The U.S. Veterans Health Administration also released data which found a much larger decrease in hospital-onset MRSA infections as opposed to hospital-onset MSSA using various infectious disease bundles that all included universal MRSA surveillance and isolation for MRSA carriers. These results mirror the results obtained by the United Kingdom’s National Health Service. These findings support the contention that the marked decline in hospital-onset MRSA infections observed in these studies is due to interventions which are specifically targeted towards MRSA.

A case is made that concerns with the integrity of healthcare policy research, along with industrial conflicts-of-interest have inhibited effective formulation of infectious disease policy in the United States. Because MRSA has become endemic in the general U.S. population (approximately 2%), the author advocates that universal facility-wide screening of MRSA on admission be included in infection prevention bundles used at U.S. hospital.

## Background

On March 4th 2019, the Centers for Disease Control and Prevention (CDC) released a Vital Signs report regarding efforts in the United States to control *Staph aureus* and MRSA. The results were not good. In 2017, there were 119,000 *Staph aureus* infections and almost 20,000 deaths [1]. The reduction in MRSA bloodstream infections stalled between 2012 and 2016. Risk-adjusted data using the CDC’s Standardized Infection Ratio (SIR) reported an 8% reduction in 2017 compared to 2016 [2]. However, there is still wide variation in performance [3]. The United States is nowhere near on track to meet the 2015 U.S. Dept of Health and Human Services goal of a 50% reduction by 2020 [4]. It is the purpose of this commentary to review the formulation and history of MRSA control policy in the United States with emphasis on the role of carrier surveillance, along with policy implications of the newly released CDC data and Veterans Health Administration MRSA infection control report..

### Implications of differing control rates for MRSA and MSSA

In the Vital Signs report, [1] the CDC did not make many new recommendations. However, they did highlight the large decrease in MRSA which was achieved by the U.S. Veterans Health Administration. The report observed a 66% decrease in hospital-onset MRSA, but only a 19% decrease in MSSA [5]. The emphasis was that improvement can take place, but what was not said was even more important.

The relatively small decrease observed in MSSA strongly supports the contention that strategies specifically directed at MRSA, as opposed to global strategies were responsible for the large fall in MRSA infections. In Veterans Health Administration, this comprised universal surveillance of all admitted patients and isolation of carriers. A similar observations was also found buried in the Electronic Medical Record data in the Vital Signs Report [1]. Hospital-onset MRSA bloodstream infections showed a significant decrease but hospital-onset rates of MSSA were unchanged.

All of this was foretold by a 2017 ARIC article which reported that rates of hospital-onset MRSA bloodstream infections were not falling in the United States and suggested that a reexamination of the Veterans Health Administration MRSA infection data might hold lessons on how to reduce MRSA infection rates [6]. 

We have identified a total of four reports which have observed a marked decrease in hospital-onset MRSA infections and little or no decease hospital-onset MSSA infections.

1. United States - Veterans Health Administration [5].
2. United Kingdom - National Health Service (NHS) [7, 8].
3. Seville, Spain - Jesus Rodriguez-Bano, et al [9].
4. United States - CDC Electronic Medical Record Data from 447 hospitals [1].

In these four reports one may conclude that strategies designed specifically for MRSA must have been the driver in the observed reduction in MRSA infections. Infectious disease prevention strategies used in the first three reports included surveillance on admission for MRSA carriers with implementation of isolation and/or decolonization. Infectious disease prevention protocols were not described in the fourth report. In the United States private sector, Rodriguez-Bano, et al., the VA and NHS also implemented protocols to promote hand hygiene. However, if hand hygiene was the driver in hospital-onset MRSA reduction, we should also have observed a reduction in hospital-onset MSSA.

### How did the United States largely abandon surveillance

The main interventions in the United States to stop the MRSA epidemic have not centered on surveillance and isolation / decolonization. Two papers, Harbarth, et al., published in JAMA [10] and Huskins, et al., published in NEJM [11] have been used by policymakers to not recommend the expanded use of surveillance and isolation [12–16]. In addition, there has been a demand for rigorous studies after a review by Cooper, et al., found that most surveillance studies were not well controlled. [17, 18] This philosophy continued despite Harbarth, et al., and Huskins, et al. being criticized for major design flaws [19, 20] and a major controlled study demonstrating the efficacy of universal facility-wide surveillance in decreasing MRSA infections [21].

### Research integrity issues with underlying health policy research

In the United States, hand hygiene has been one of the cornerstones of infectious disease policy. However, its effectiveness as a primary intervention appears to have been overstated, as illustrated by at least one article [22]. In this instance, industrial conflicts of interest appear to exist at both the research and editorial level [23]. Hand hygiene is an important part of an infectious disease control bundle, but as supported by the results in the above four research reports, targeted pathogen specific interventions are needed to markedly decrease infection rates.

Universal use of chlorohexidine has also been advocated and has distracted some institutions in their efforts to control MRSA. The basic research behind widening the applications of chlorhexidine has been riddled with research integrity problems and industrial conflicts of interest. Numerous research papers have performed two-to-one comparisons, comparing the efficacy of chlorhexidine plus alcohol to alcohol alone [24]. Industrial conflict of interest was brought to a head by the Charles Denham scandal which involved the National Quality Forum, a non-profit organization in the United States that advises CMS on quality measurements and patient safety indicators [25].

A major report on the efficacy of universal daily chlorhexidine bathing in the ICU [26] had debatable conclusions, showing a non-significant decrease in MRSA bloodstream infections with the most significant effect on commensal bacteria and yeast. This report also appeared to have issues of research integrity and spinning [20] and was the subject of a Reuters Investigative report which found apparent industrial conflicts of interest. [27] On a facility-wide basis, universal chlorhexidine bathing was found not be effective in the prevention of MRSA [28, 29]. However, certain high-risk patients with medical devices may benefit from this intervention [28].

### Interventions which currently are receiving emphasis in the United States

Despite the mounting evidence for the need for pathogen-specific targeted interventions, many institutions in the United States continued to exclusively implement non-specific global interventions. These have included:

1. Hand hygiene. Hand hygiene is extremely important. It is the “plastic straw” of infection control. But by itself it is of questionable value. In the context of MDROs, hand hygiene should be viewed as a backup measure, since these organisms should not be on a healthcare workers’ hands in the first place. And if they are, there is a problem with containment and control.
2. Focusing on the farm and not healthcare. The justification for this argument is that even though rates of usage are dropping, almost 11 million kg of antibiotics were consumed by U.S. farm animals in 2017 [30]. This argument has been used in an attempt to bolster risk adjustment for hospital-onset MRSA infections using community levels of MRSA infections. However, recent epidemiological research from the European Union has brought the concept of agriculture antibiotic usage as a major driver of human infections into question [31]. And one must ask, if MRSA is so prevalent in the community, regardless of the source, why are institutions not screening all patients upon admission?
3. Antibiotic stewardship. This is extremely important, but there is no guarantee this will reverse the current epidemic or stop new resistant organisms from developing. Even if usage is cut by 50%, there will still be billions of bacteria exposed to antibiotics and resistance may still develop, but hopefully at a slower rate. In addition, antibiotic stewardship’s efficacy in stopping an epidemic caused by endemic pathogens may differ from its efficacy in preventing future epidemics from emerging drug-resistant pathogens.
4. Universal daily bathing with chlorhexidine. Because of the history of research integrity problems surrounding this product, [16, 24] along with the recent well-controlled study showing its lack of effectiveness in preventing MRSA infections on a facility-wide basis (with the exception of patients with medical devices), [28] we agree with Olivier Mimoz and Jérémy Guenezan who stated in a recent Lancet commentary that “Chlorhexidine use should consequently be limited to situations presenting a clear patient benefit” [29].

### Common excuses for the high rates of hospital-onset MRSA in the United States

There are a number of common excuses which are used to justify the high rates of hospital-onset MRSA and the inertia in implementing protocols to lower infections.

**Fig. 1:**
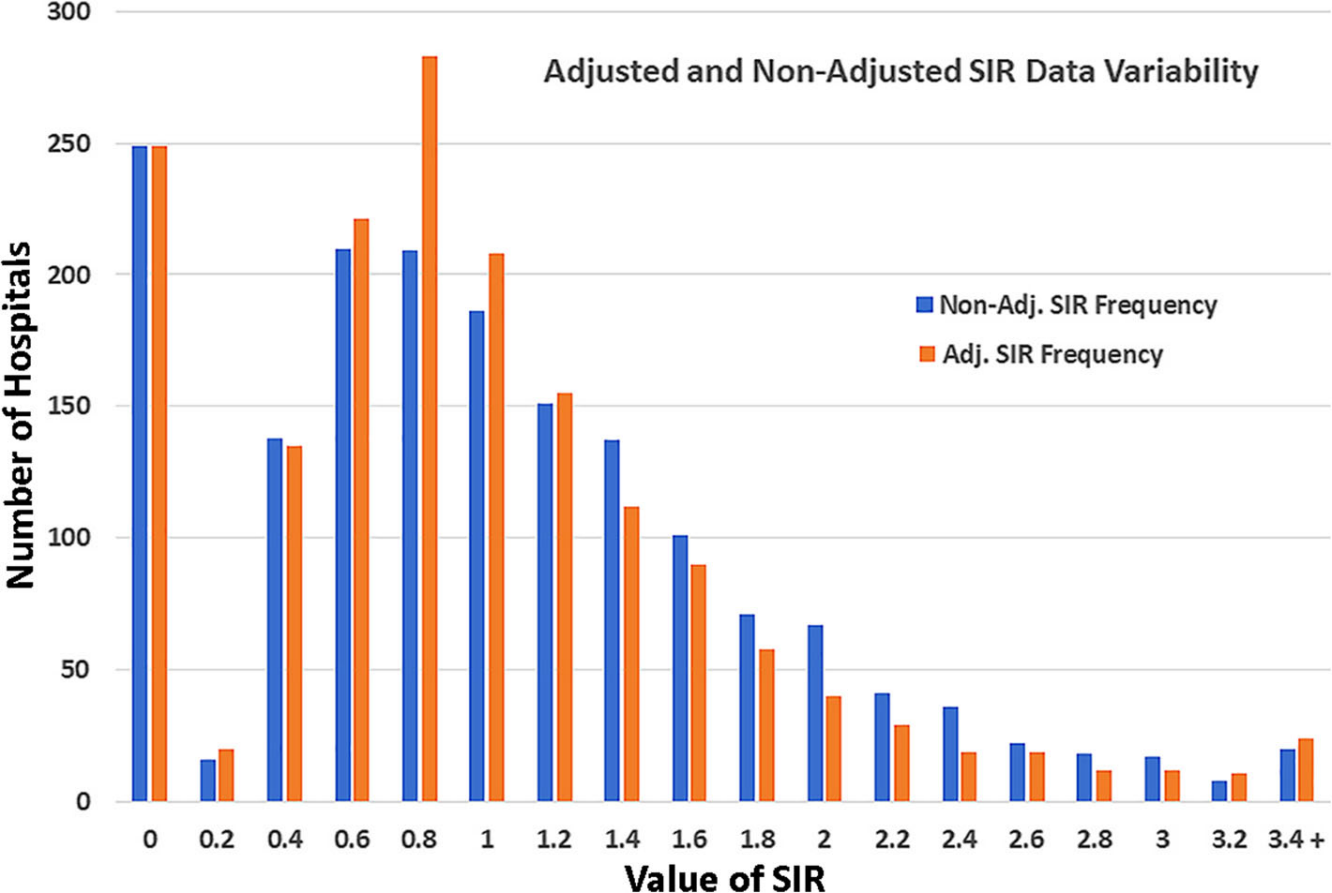
Risk Adjustment Data Variability in the Standardized Infection Ratio. Acquisition Dates 1/4/2017 to 31/3/2018, 1697 hospitals analyzed

1. Citing the epidemic of illicit opioid injection injecting opioids for the increased in community MRSA and for the increase risk of obtaining a hospital acquired infection, has become another common excuse to justify inaction. There are calls by the industry to increase risk adjustment because of the increase risk of infections caused by the opioid epidemic. However, the proportion of total MRSA infections represented by patients who inject opioids only increased by 5% (4% in 2011 to 9% in 2016) [32]. Thus, one could argue that instead of risk adjusting the effects of the opioid epidemic away, United States’ facilities should place more emphasis on screening and isolation / decolonization.
2. Citing the high rate of MRSA in the community. Similar to the opioid epidemic, high rates of MRSA in the community have increased calls for risk adjustment of hospital acquired infection rates. Currently, risk adjustment can decrease the reported Standardized Infection Ratio (SIR) or adjusted infection rate of a facility by over 50% (see Table 1). Instead of improving reported performance by mathematically lowering infection rates with the SIR, a better strategy would be to implement pathogen specific interventions, such as surveillance and isolation, to actually decrease infections which are associated with community environmental pressure. This type of risk adjustment also causes an aberration of increasing the SIR in some “low risk” facilities. The overall effect is to decrease interfacility variability (see Fig. 1) and disincentivizes support for infection control. Thus, the concern is that risk adjustment is not adjusting only for patient risk but for facility underperformance.
  Similarly, changing the grace period for the diagnosis of healthcare associated hospital acquired MRSA bloodstream infections in the United States from 2 to 3 days lowers the number of infections reported. The smaller numbers make interfacility differences less likely to reach statistical significance, resulting in more facilities being designated as “No Different Than National Benchmark”.
3. Citing the staff’s hand washing compliance is also used to justify inaction. The reason given is that if we cannot get the staff to reliably wash their hands, then everything else is unlikely to work. There is no significant evidence that destroying the microbiome on a healthcare workers hands 100 times a day is a prerequisite to infection control. Few if any centers have been able to reliably accomplish this. However, the above four reports showing a marked decrease in hospital-onset MRSA as compared to hospital-onset MSSA present a strong combined argument that targeted infection control can be effective, even if hand hygiene compliance is not 100%.
4. Believing that decolonization cannot impact the health of a patient or healthcare worker who is colonized with MRSA. However, decolonization can be effective for both healthcare staff and patients. Albrich, et al., reported that 88% of 510 healthcare workers were successfully decolonized for MRSA [33]. Huang SS, et al., in a recent NEJM article, reported a well-controlled large prospective study (Project CLEAR) which found that outpatient decolonization of patients after hospitalizations can significantly decrease infections. [34] The World Health Organization recommends that all presurgical patients undergo surveillance for *Staph aureus*, along with decolonization. [35] However, in the United States, this has yet to become a standard of care, not even for the most dangerous form of *Staph* aureus, MRSA.

**Table 1:** MRSA Bloodstream Infections in US Hospitals Having Major Risk Adjustment. Data Acquisition Dates 1/4/2017 to 31/3/2018

Hospital Name	City	State	Patient Days	Risk Adj. Perdicted Cases	Non-Risk Adj. Perdicted Cases*	Observed Cases	Risk Adj. SIR	Non-Risk Adj SIR*	Percent difference**
HOSPITAL FOR SPECIAL SURGERY	NEW YORK	NY	50,032	1.148	2.606	1	0.871	0.384	127.01%
HIALEAH HOSPITAL	HIALEAH	FL	54,002	1.348	2.813	6	4.451	2.133	108.69%
MAGEE WOMENS HOSP. OF UPMC HEALTH SYSTEM	PITTSBURGH	PA	94,175	2.461	4.906	3	1.219	0.612	99.35%
WOMEN & INFANTS HOSPITAL OF RHODE ISLAND	PROVIDENCE	RI	77,556	2.082	4.040	2	0.961	0.495	94.13%
WOMANS HOSPITAL OF TEXAS,THE	HOUSTON	TX	110,317	2.984	5.747	2	0.670	0.348	92.52%
MEDSTAR GEORGETOWN UNIVERSITY HOSPITAL	WASHINGTON	DC	121,973	13.276	6.354	10	0.753	1.574	−52.15%
PENN PRESBYTERIAN MEDICAL CENTER	PHILADELPHIA	PA	90,041	9.801	4.691	6	0.612	1.279	−52.16%
UNIVERSITY HEALTH SYSTEM	SAN ANTONIO	TX	180,652	19.662	9.411	12	0.610	1.275	−52.16%
UNIVERSITY OF IOWA HOSPITAL & CLINICS	IOWA CITY	IA	213,095	23.193	11.101	11	0.474	0.991	−52.16%
INDIANA UNIV. HEALTH BALL MEMORIAL HOSPITAL	MUNCIE	IN	88,914	9.678	4.632	4	0.413	0.864	−52.18%

A smaller SIR denotes better performance

*Estimated using total number of U.S. MRSA Bloodstream Infections and total number of U.S. Hospital Patient Days

**Negative values increases performance with a smaller SIR, postive values decreases performance with a larger SIR

Non-Risk Adjusted SIR was calculated using a ratio of the hospital’s observed cases / the hospital’s Non-Risk Adjusted Perdicted Cases

Non-Risk Adjusted Perdicted Cases was calucated by multiplying the Non-Risk Adjusted National Infection Rate by the number of facility Patient Days

Non-Risk Adjusted National Infection Rate equals the the sum of the national total Observed Cases divided by the national total number of Patient Days

N equaled 3917 U.S. facilities

### Need for transparency and more comprehensive reporting

The healthcare industry in the United States is not making the hard decisions and allocation of resources needed to reverse this epidemic. The industry has largely avoided the issue of identification and reporting of colonized patients and healthcare workers, along with the risk these colonized pathogens pose to their health, and the health of their families and patients.

In addition, the United States has changed metric definitions [36] and uses stringent risk adjustments for reporting MRSA along with other hospital acquired infections. This has the potential of severely mitigating the actual numbers of patients. In the historical review “The Pandemic Century” [37], it is apparent this strategy has been used in many past pandemics, with governments trying to avoid accountability. For example: During the 2014 Ebola epidemic, the government of Guiana insisted that only laboratory confirmed cases be counted. In a country with sparse medical resources this created an underestimation of cases and the appearance that the epidemic was being brought under control, greatly delaying the international response and setting the stage for the carnage that followed. And, in the 2015 Zika epidemic in Brazil, the definition of microcephaly was changed from a head circumference of 33 cm to 32 cm which reduced the number of cases.

When risk adjustment is performed, it should be based on high performance facilities which have optimally implemented preventative strategies. However, when this is done, even with high risk populations as serviced by the Veterans Administration, MRSA infection rates have been observed to fall to an extremely low level [38] questioning the need for adjustment. The same is true for Central Line Bloodstream Infections (CLABSIs) [39].

## Conclusion

In the face of an emerging epidemic, medicine must not stagnate by waiting for the performance of randomized controlled trials(RCT). This is what appears to have happened in the United States and in the review by Cooper, et al. [18] was used as justification [17]. However, epidemics evolve faster than randomized controlled trials can be planned, approved and conducted. Conditions change. In the United States, the epidemic of MRSA has progressed becoming endemic in its population. Not since the Spanish Flu Outbreak of 1918 has an epidemic of this magnitude existed in the United States and similar tactics may need to be adopted.

It is known how to control the spread of MRSA in acute care facilities. The use of facility-wide admission screening is a strategy reminiscent of reverse isolation used in the Spanish flu epidemic, where a barrier is placed between the community and the facility to protect the most vulnerable of individuals, patients in hospitals. The VA has had great success with this strategy, it now needs systemwide implementation in the United States. Additional measures which should be enacted include instructing visitors regarding good hygiene and the prohibition of young children from visiting a facility, both from the standpoint of safety of the child and patients. The use of daily Chlohexidine bathing is associated with concerns of fostering resistance [40, 41] and on a facility wide basis it has not been shown to be effective in preventing MRSA infections, except in patients with medical devices [28].

Control of MRSA in the community is another issue. MRSA is endemic in the United States, with the CDC estimating a 2% rate of carriage in our general population. It is known that this carriage carries a greater risk of infections, [34, 42] many of which can be prevented by decolonization [34]. Households have been shown to be an important reservoir for MRSA, where it may persist for 2 to 8 years and decolonization of household members may be an important component of an MRSA control strategy [43]. Additional research is needed on how to best decolonize citizens, the best ways to clean the environment and how to prevent reconversion.

The cost will be high. For example, Project CLEAR took approximately 8 years from commencement to publication [44] at a cost of almost 10,000,000 USD from AHRQ funding alone [45]. The United States Government has limited resources to fund these large studies, especially when strapped with funding the most expensive healthcare system in the world [46] where the average CEO‘s salary at 22 major non-profit hospitals is 3.1 million dollars per year [47]. Obviously, we cannot wait for RCT design and completion before taking decisive action to confront epidemics of emerging and everchanging drug resistant bacteria.

The United States needs to regain its leadership role in the World in confronting these pathogens, which pose both a national and international risk and not fall into the trap of avoiding setting standards by using the excuse of “One size does not fit all”. The lack of firm standards has also led to an almost lackadaisical attitude in the control of dangerous pathogens with some U.S. facilities viewing MRSA carriage as “no big deal” [48] and not even placing patients who have MRSA infections or colonizations in full contact precautions .

The principal deputy director of the CDC was quoted by USA Today as stating the stalling in the reduction of MRSA infections in the United States might indicate that healthcare facilities are “wondering whether it’s worth their trouble” to take action against these dangerous pathogens [49]. First and foremost, the United States must regain control of its fractured and disparate healthcare system. Uniform action must now take place. Similar to the Plague outbreak in San Francisco in 1924, the United States government may be able to obtain limited statutory authority to mandate uniform reporting of highly dangerous pathogens and oversee containment and control by using the 2005 WHO International Health Regulations [50], which arguably could be applied to any State with an air or sea port of entry.

The United States has the knowledge and resources, but it has yet to prove it has the political and economic will to contain this epidemic.

## Data Availability

Data can be downloaded from Hospital Compare datasets. Data.Medicare.gov. Mar. 21, 2019. https://data.medicare.gov/data/hospital-compare Accessed on Mar. 23, 2019.

## Abbreviations

ARIC: Antimicrobial Resistance and Infection Control
CDC: Centers for Disease Control and Prevention
MRSA: Methicillin-resistant *Staphylococcus aureus*
MSSA: Methicillin-sensitive *Staphylococcus aureus*
NHS: National Health Service
SIR: Standardized Infection Ratio
